# The movement and deposition of PM2.5 in the upper respiratory tract for the patients with heart failure: an elementary CFD study

**DOI:** 10.1186/s12938-016-0281-z

**Published:** 2016-12-28

**Authors:** Tiantian Zhang, Bin Gao, Zhixiang Zhou, Yu Chang

**Affiliations:** School of Life Science and Bioengineering, Beijing University of Technology, Beijing, 100124 People’s Republic of China

**Keywords:** PM2.5, Heart failure, Aerodynamics, Particle deposition, Wall shear stress

## Abstract

**Background:**

PM2.5 is an important factor to affect the patients with respiratory and cardiovascular diseases. Clinical studies have found that the morbidity and mortality of patients with heart failure (HF) have a close relationship with the movement and deposition state of PM2.5. One reason is that the breathing pattern of patients with HF has obvious difference with healthy people, however the effect caused by these differences on the distribution regularity of PM2.5 in the respiratory tract is still unclear. Hence, a computational fluid dynamics simulation was conducted to clarify the aerodynamic effect of breathing pattern of patients with HF on respiratory system.

**Methods:**

Ideal upper respiratory tract geometric model was established based on standardized aerosol research laboratory of Alberta and Weibel A dimension. The discrete phase method is used to calculate the movement of the airflow and particles. The flow rate were chosen as the inlet boundary conditions, and the outlets are set at a constant pressure. The rate of particle deposition, distribution location, wall pressure, flow velocity and wall shear stress are obtained, and compared to the normal control.

**Results:**

The results demonstrated that the rate of escaped particles in every bronchial outlet of the patients with HF was more than the normal controls, meanwhile the trapped was less (1024 < 1160). There was higher by 12.9% possibility that the PM2.5 entered the lungs than the normal control.

**Conclusion:**

The aerodynamic performances of HF patients are different from normal control. Compared to the normal control, under similar environment, there is higher possibility of PM2.5 moving into lungs, and these particles could affect the function of the respiratory system, resulting in the deterioration of the state of cardiovascular system. In short, it’s necessary to pay more attention to the living environment of HF patients, to reduce the content of PM2.5 particles in the air, and reduce the damage of PM2.5 particles caused by breathing patterns.

## Background

Heart failure (HF) is the end stage of cardiovascular disease [1], including coronary heart disease, rheumatic heart, and high blood pressure [2–4]. However clinical studies have found that the breathing pattern of HF is distinguishing with the normal person [5].

Meanwhile, with the increase of awareness of air pollution, PM2.5 particles on the impact of cardiovascular patients have also received more and more attention. PM2.5 refers to the micron particles whose equivalent diameter is less than or equal to 2.5 µm, accounting for more than 74% of the total air pollution particles. Because of the large particles surface area, small particle diameter, and containing a large amount of harmful toxic characteristics, PM2.5 can follow breathing into the human body, and even reach the alveoli and in the blood [6–9]. So the damage of PM2.5 to the lung is obvious and direct, moreover its effects on the heart become more and more important for the researchers in recent years. Epidemiological investigation showed that PM2.5 is associated with morbidity and mortality of heart failure. The concentration of PM2.5 rises 3 µg/m^3^, the number of heart failure patients increases by 4.70% [10–14]. Thus, PM2.5 pollution and the morbidity and mortality of heart failure has certain relevance. But its mechanism is still not clear.

Experimental results show that the influence of atmospheric pollutants on the heart failure, on the one hand is because of some components of PM2.5 directly into the circulatory system induced the formation of blood clots, and impact on the cardiovascular system [6, 15]; on the other hand, PM2.5 can produce inflammation and stimulate respiratory tract release cytokines, cause blood vessel damage, leading to thrombosis, influence the stability of atherosclerotic plaques [7–9]. British and Indian joint research shows that air pollution is closely related to the hospitalization rate and mortality rate of heart failure. The researchers selected 35 relevant researches to study the correlation of pollution particle concentration and hospitalization or death rate. PM2.5 concentrations increase 10 µg/m^3^, rate of hospitalization and mortality increased by 2.12%.

Pope C A assessed the relationship between air pollution and the admission and mortality rate of decompensated heart failure, based on a systematic review and meta-analysis of the previous research. Results show that the air pollution and heart failure hospitalization rate and mortality has a close correlation [6]. Meanwhile, it has a strong correlation between the time length of exposure to air pollution and the risk of hospitalization and death caused by heart failure. The longer the exposure time is, the greater the impact of the continuing role of PM2.5.

Research shows that the movement and deposition of the air pollution particles in the upper respiratory tract are closely linked to the respiratory rate, vital capacity parameters [18, 19]. The computational fluid dynamic (CFD) as an effective method were widely used to evaluate the aerodynamic effects of several diameters of particles on respiratory tract. Benjamin [16] found that under different constant speed respiratory rate, the movement and deposition of particles in the upper respiratory tract have obvious difference. Clement [17] studies have found that under the same respiratory rate, the movement and deposition of different particle diameter are also different. Zhang [18–24] had calculated the movement and deposition of PM2.5 particles in the upper respiratory tract when the normal human respiratory rate is constant (15–60 l/min) by using the method of fluid dynamics of discrete phase. The calculation results show that the particle deposition rate and respiratory flow rate and particle diameter are correlated, the deposition rate is higher in the throat and tracheal bronchus bifurcation, and with the increase of respiration velocity, particle deposition rate will decline in the upper respiratory tract, so the possibility of getting into the lungs will be higher. Cheng [25] researched the effects of particle size and respiratory rate on the deposition results in human oral respiratory tract by comparing with nine different diameter of particles and the conditions of three kinds of respiratory rate. The result implied that the smaller particle' diameter and the higher respiratory rate will cause the lower deposition rate and the higher escaped rate. Vasconcelos [27] calculated the particle escape rate and capture rate in the tertiary bronchi branch by using the method of computational fluid dynamics. Li [24, 26] adopted the approach of multiphase flow, and sets the particles to the discrete phase, air to continuous phase. So he made use of the real human bronchial model to calculate the movement of micron particles, and got the results of the particle deposition position. Furthermore, he put forward that the real breathing entry conditions could be set as the further work optimization.

As we can see from the above several studies, CFD has been used to model airflow, temperature and particle deposition within the human upper respiratory tract [15–25]. In these studies, researchers adopted constant flow to simulate the fluid dynamics movement of particles in the respiratory system, however gas flow rate, the frequency of breathing, tidal volume, inspiratory time accounts for the proportion of the respiratory cycle index did not study. Different breathing rate and tidal volume will significantly change the fluid dynamics inside the upper respiratory tract environment, may affect the movement of PM2.5. But the upper respiratory tract in patients with heart failure in the influence of the particle movement law is not clear. So the movement of PM2.5 particles in patients with heart failure of the upper respiratory tract and deposition state is necessary.

In order to clarify this problem, numerical studies were conducted. Ideal upper respiratory tract models with three levels bronchi were established based on physiological data. The breathing flow rate, derived from previous work, were chosen as the boundary conditions. The flow pattern in the respiratory tract, the trapped rate ratios of particles, the position of particles deposition, flow interface, and wall shear stress (WSS) were used as the factors to evaluate the hemodynamic states.

## Methods

### Geometric model

The upper respiratory tract ideal model was constructed by using a commercial software *SolidWorks14.0* (Solidworks Corp., MA, USA), referred to the standardized ARLA [28] and Weibel A [29] model dimension (Table 1). The size is measured by screening of three volunteers without a history of acute and chronic upper respiratory tract disease.

**Table 1:** Statistics of key dimensions of the upper respiratory tract

Key parts	ARLA model and Weibel A model		Angle (°)
	Diameter (mm)	Length (mm)	
Soft palate	27*15	24.1	–
Uvula	22.7*14	6.8	–
Epiglottis	31.2*15	39.8	–
Trachea	14.3	267.4	0
Left main bronchus	8.7	50.6	25
Right main bronchus	13.1	22.2	50
Left secondary bronchus	9	12.9	37
Right secondary bronchus	8.9	16.3	37
Left third bronchus	6	9.3	37
Right third bronchus	6.4	11	37

**Fig. 1:**
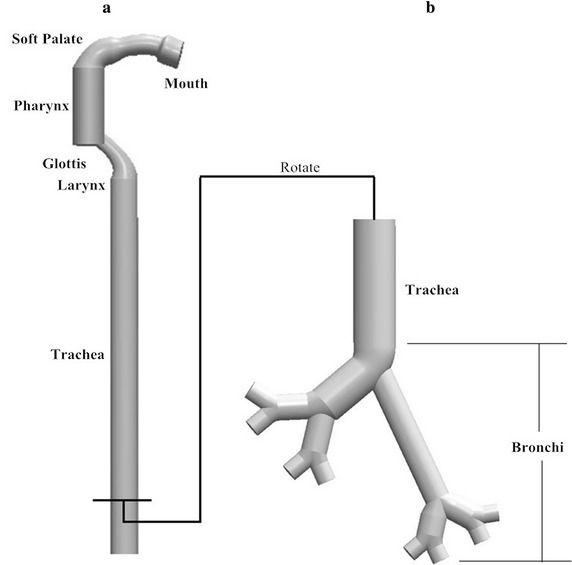
The ideal human upper respiratory tract model. *VT* tidal volume, *VE* minute ventilation, *T1* inspiratory time, *Ttot* total respiratory time, *VT/T1* mean inspiratory flow, *bpm* breaths per minute.** a** The lateral view of the parts abive trachea.** b** The front view of the bronchi

These structures are integrated according to the dimension, so it can obtain the four-generation human airway model (Fig. 1) that has been widely applied [18–24]. Figure 1a, b is the model of parts before trachea and bronchus, respectively. The model consist of the soft palate, the uvula, the epiglottis, the trachea and three level bronchus, and it can assume that the upper respiratory tract structure of heart failure patients had no obvious change.

### CFD simulation and boundary conditions

#### Transport equations

The air flow of upper respiratory tract calculation is based on three-dimensional incompressible Navier-Stokes equation (1) and (2):

1 $$\rho \frac{{\partial \vec{v}}}{\partial t} + \rho (\vec{v} \cdot \nabla )\vec{v} = - \nabla p + \mu \nabla^{2} \vec{v}$$

2 $$\nabla \cdot \vec{v} = 0$$

where *ρ* stands for the density of air, $\vec{V}$ is the fluid velocity vector, *p* is the pressure, *µ* is the dynamic viscosity.

The orbit of discrete phase particles is solved by particles force differential equation of integral Laplace coordinates. The force equilibrium equation of particles in Cartesian coordinates (x direction) is:

3 $$\frac{{dv_{p} }}{dt} = F_{D} (v - v_{p} ) + \frac{{g_{x} (\rho_{p} - \rho )}}{{\rho_{p} }} + F_{x}$$

where *F*_*D*_(*v* − *v*_*p*_) stands for unit mass drag of particles, *g*_*x*_ is the gravity in x direction, *F*_*x*_ is the other forces acting on the particles. For a diameter of 1–10 microns, *F*_*D*_ is defined as:

4 $$F_{D} = \frac{18\mu }{{d_{p}^{2} \rho_{p} C_{c} }}$$

where *v* is the fluid velocity, *v*_*p*_ is the particle velocity, *µ* is the dynamic viscosity, *ρ* is the fluid density, *ρ*_*p*_ is the particle density, *d*_*p*_ is the particle diameter.

Drag coefficient is Cunningham correction of Stokes’ drag force formula, the calculation formula is:

5 $$C_{c} = 1 + \frac{2\lambda }{{d_{p} }}(1.257 + 0.4\,e^{{ - (1.1d_{p} /2\lambda )}} )$$

where *λ* is the mean free path of gas molecules.

The particle diameter set in the calculation (1–2.5 µm) match the conditions of Eq. 5 (1–10 µm), so the Saffman lift need to be considered in the calculation. The general expression of the lift is:

6 $$\vec{F} = \frac{{2 \times 2.594v^{1/2} \rho d_{ij} }}{{\rho_{p} d{}_{p}(d_{lk} d_{kl} )^{1/4} }}(\vec{v} - \vec{v}_{p} )$$

where *d*_*ij*_ is the fluid deformation rate tensor.

The escaped rate and particle deposition rate is an important index to show the movement state of the particles, and the escaped rate is defined as

7 $$R_{E} = \frac{{N_{1} + N_{2} + \cdots + N_{8} }}{{N_{0} }}$$

where $\left\{ {N_{1} ,N_{2} , \ldots ,N_{8} } \right\}$ is the number of particles that escaped from every bronchi outlet, *N*_0_ is the number of particles that injected from the inlet surface.

In Fluent, the particle deposition rate is defined as:

8 $$R_{a} = \sum\limits_{p = 1}^{p = N} {\frac{{m_{p} }}{{A_{face} }}}$$

where N is the number of particles released from the entry of the respiratory ideal model, *m*_*p*_ is the mass flow rate of particle flow, *A*_*face*_ is the surface area of the particle collision boundary. So the adsorption of particles on the wall can be shown by deposition contour.

### Boundary conditions

Ambtosino [4] tested the ventilator pattern of 45 patients with chronic heart failure and 22 normal through the experimental measurement, and got the statistics of the mean and SD (standard deviation) of tidal volume (V_T_), respiratory frequency and inspiratory time (T_1_). There was some difference of the results, as showed in Table 2.

**Table 2:** Ventilatory pattern of HF and controls

	Controls	HF (NYHA III)
V_T_, ml	920 (155)	949 (297)
Respiratory frequency, bpm	16 (4)	18 (5)
V_E_, l/min	14.9 (4.1)	16.6 (5.5)
T_1_/T_tot_, %	43 (5)	44 (4)
V_T_/T_1_, ml/s^−1^	568 (147)	622 (205)

Mean (SD); *NYHA* New York heart association, *V*_*T*_ tidal volume, *V*_*E*_ minute ventilation, *T*_*1*_ inspiratory time, *T*_*tot*_ total respiratory time, *V*_*T*_ */T*_*1*_ mean inspiratory flow, *bpm* breaths per minute

**Fig. 2:**
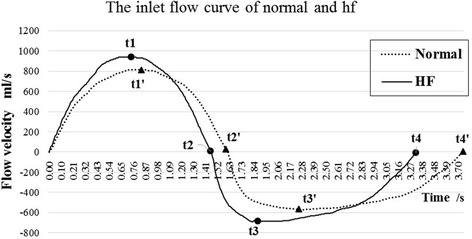
The flow-time curve of normal and heart failure patients. The eight marked points (*t1*,* t2*,* t3*,* t4*, and* t1′*,* t2′*,* t3′*,* t4′*) on the* two curve* are four representative times respectively

Human unsteady breath flow-time curve can be measured by using NR—6 nasal sound reflection instrument. According to the index of V_T_ and T_1_ in Table 2, the Fourier transform was carried out on the flow-time curve to change the timeline and amplitude. So the flow-time curve of normal and NYHA III heart failure patients can be got, as shown as Fig. 2. The bronchi outlets are set at a constant pressure of 1.0 atm.

### Numerical methods

**Fig. 3:**
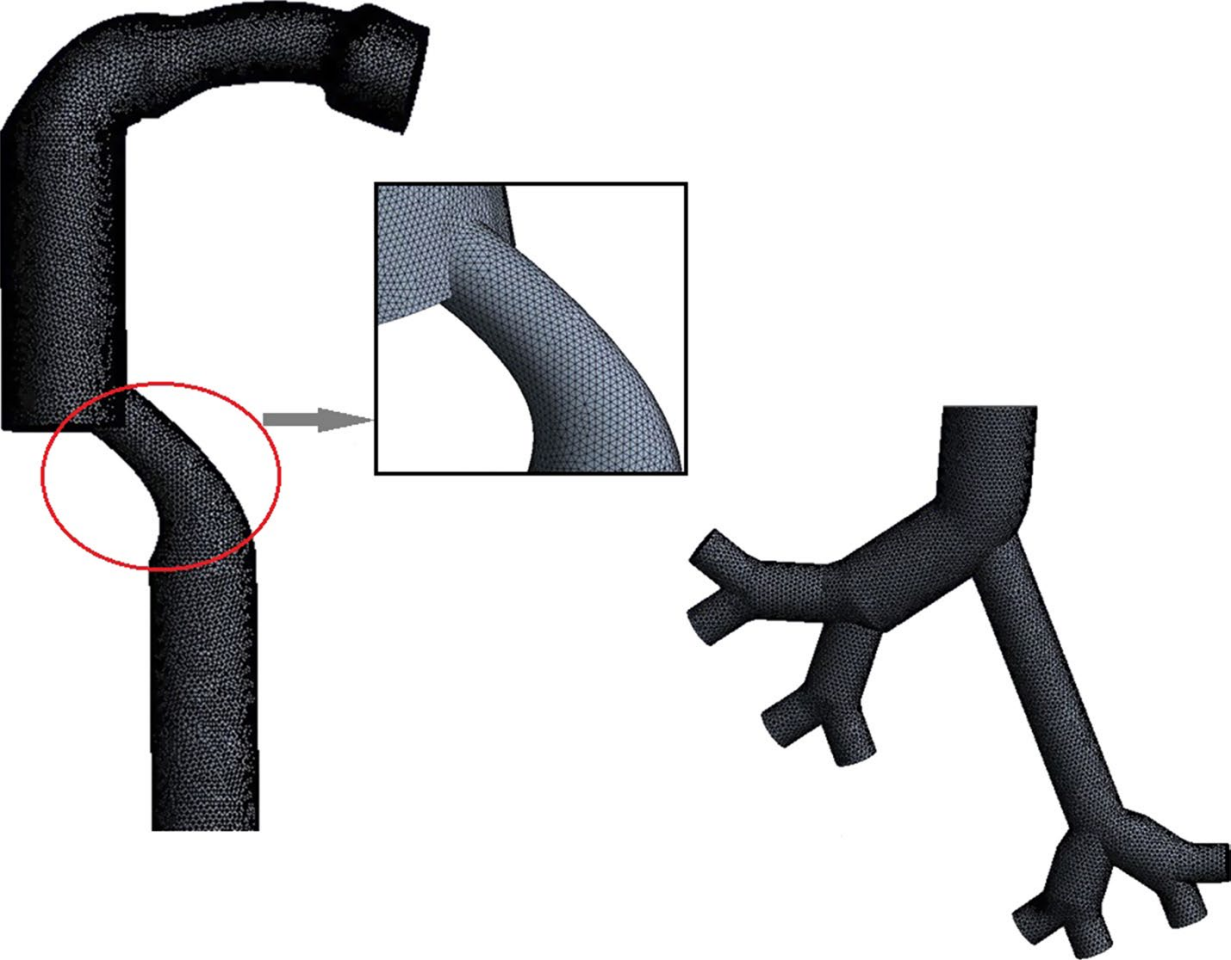
The mesh result of the ideal human upper respiratory tract model

The modelled upper respiratory tract ideal model was read into the CFD solver, ANSYS Fluent 14.5(ANSYS Inc., PA, USA). And tetrahedron elements are used during the meshing process. The base size of the mesh is 0.2 mm but the final size is determined through mesh-independent evaluation with a tolerance of <0.3%. Depending on the size of the model, 1017,289 elements are included. Further the grid independence test is conducted to determine the optimal grid numbers for the computation. The test result was shown as Table 3. According to the results, 1.01 million elements are enough for this study. The mesh is shown as Fig. 3.

**Table 3:** The grid independence test

Nodes	Elements	R_T_ (%)	Relative error (%)
107270	545774	78.4	4.21
194088	1017289	81.7	1.22
336768	1768311	82.7	

*R*_*T*_ the rate of trapped particles

The flow-time boundary conditions are imported in the Fluent 14.5. All solid boundaries on the airway surfaces were specified as rigid, no-slip walls. And it was assumed that all particles can be trapped by the wall. Correspondingly the inlet velocity reaches value as high as the low-Reynolds-number (LRN) standard *k*−*ω* turbulence model was applied to the domain with a turbulence intensity of 5% specified for all flow boundaries and for initialization. The turbulence model has previously been used in respiratory flow simulations [16–27].

The total flow through the model consisted of two components, standard air and carbon (C) particles, the highest percentage of the composition caused by air pollution. The air was modelled as an ideal gas with a dynamic viscosity of 1.7894 × 10^−5^ kg/(m s) and density of 1.225 kg/m^3^, while C particle was modelled with a constant density of 2000 kg/m^3^ and diameters of 0.8, 1.2, 2.5 µm. The model was solved isothermally with a total condition temperature of 298.15 K as it was assumed the thermal effects on the flow were negligible.

## Results

### The deposition and escaped of PM2.5

**Fig. 4:**
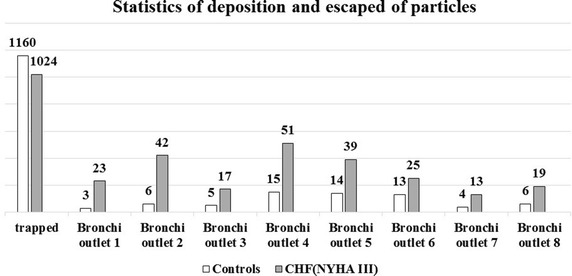
Statistics of deposition and escaped of particles

Figure 4 was the statistics of deposition and escaped of PM2.5. For the patients with heart failure, there are 18.3% of PM2.5 that would move into next level bronchi, even reach the lungs. Compared to the normal control, R_E_ is higher by 12.9% than the normal control. The statistics indicated that the number of escaped particles in every bronchial outlet of the patients with HF are more than the normal controls, meanwhile the trapped particles were less (1024 < 1160). That is, in this study, there was higher possibility that the PM2.5 entered the lungs via the upper respiratory tract of the patients with HF than the normal controls. The virus carried by particles would affect the respiratory system and cardiovascular system seriously. Then the PM2.5 aggravated the burden of cardiovascular system in the patients with HF.

**Fig. 5:**
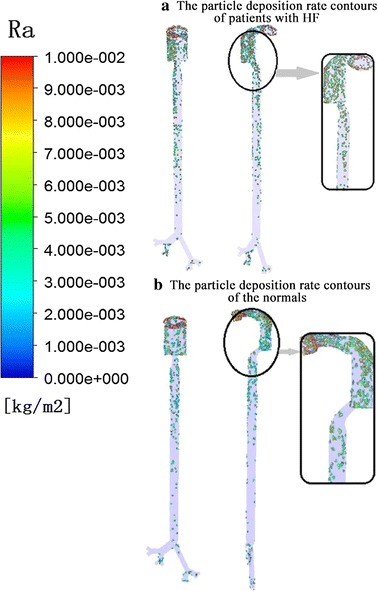
The particle deposition rate contours of patients with HF (**a**) and the normal (**b**) control

Figure 5 was the contour of the location of particles deposition. The contours represent the impact degree of the particles to the airway wall and the deposition location. Compared with the normal controls, there are more trapped particles in larynx and trachea of patients with HF (4.47 kg/m^2^). While the most particles had been trapped before the trachea. As a result of the breathing patterns of heart failure patient have the characteristics of high respiratory rate, high volume, the possibility of PM2.5 into the trachea and bronchus is greatly increased, then harmful to respiratory system and cardiovascular system.

### Wall shear stress (WSS) and wall pressure

**Fig. 6:**
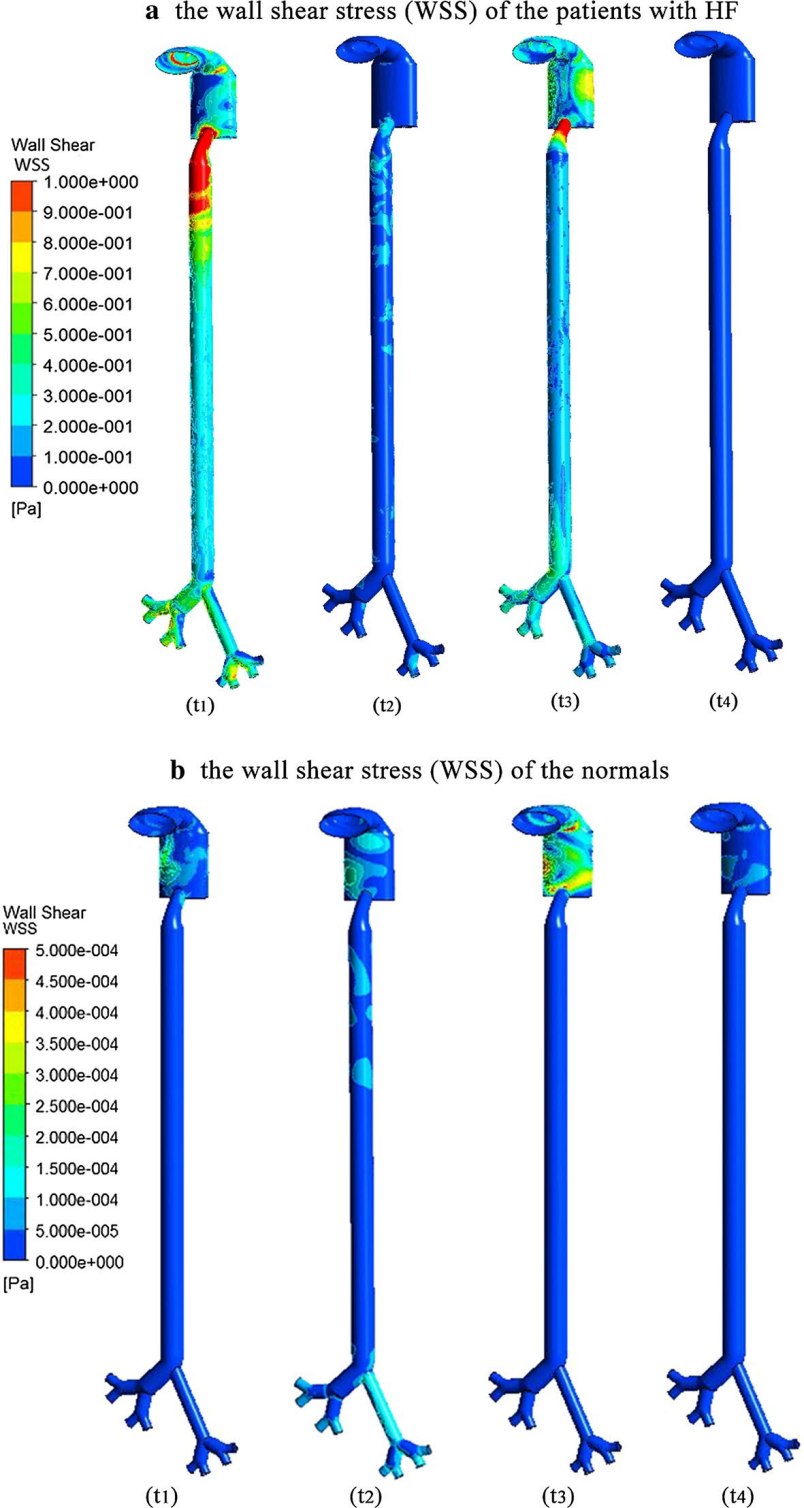
The wall shear stress (WSS) at different time of patients with HF (**a**) and the normal (**b**) control

**Fig. 7:**
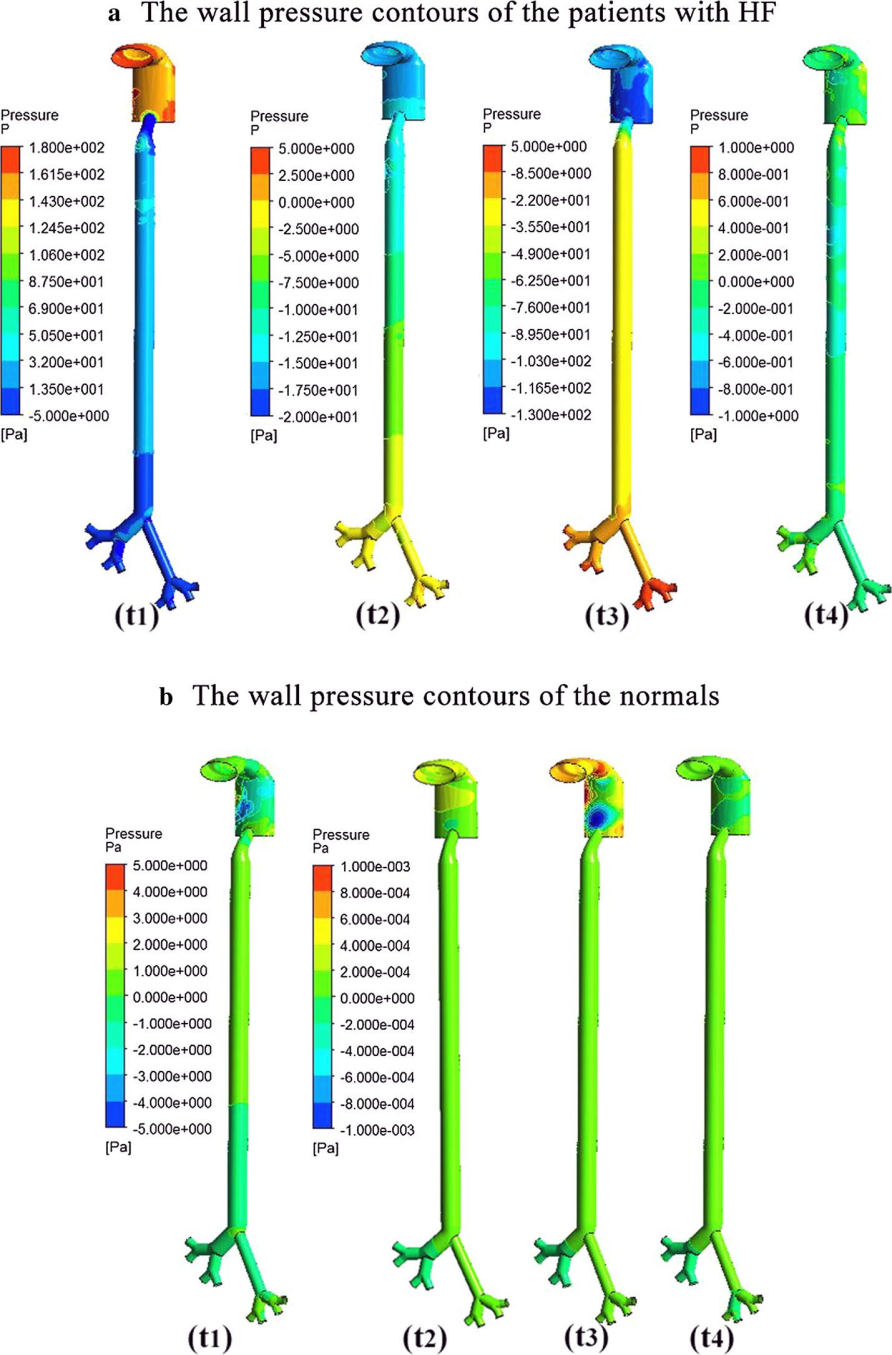
The wall pressure contours at different time of patients with HF (**a**) and the normal (**b**) control

**Fig. 8:**
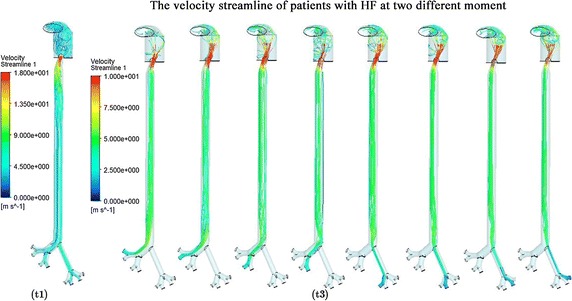
The velocity streamline of patients with HF at two different moment

Figure 6 was the contour of the shear stress (WSS) of the patients with HF (a) and the normal controls (b). Compared to the controls, the upper respiratory tract of the patients with HF suffer from higher WSS (3 vs 0.0005 Pa). The wall pressure are shown in Fig. 7. It showed that the pressure change was considerable during the whole respiratory cycle of HF than the controls (300 vs 10 Pa). As we can see from Fig. 8, the velocity streamline of patients with HF at the moment of the fastest breath in and out was showed. The air flow was linear in trachea and bronchus, and irregular flow in the throat, so less particles have been trapped by the wall. This was the main factor to effect the particles’ movement and deposition. So the upper respiratory system of HF could bear more pressure and damage. It could also induce the inflammation in upper respiratory tract.

## Discussion

HF as a severe cardiovascular disease has a higher demand on the surrounding environment, due to the particularity of body state and the seriousness of the air pollution. Although many studies focus on aerodynamic in upper respiratory tract [28–32], there is no study to link the two directions to discuss the effect of PM2.5 on the HF.

For the CFD simulation of airflow in the respiratory tract, several important assumptions are made, including highly simplified geometry, the rigid airways with smooth surface, the no-slip and absolute-adsorption boundary conditions, modeling air as an incompressible Newtonian fluid with constant density and viscosity [1.225 kg/m^3^ and 1.7894 × 10^−5^kg/(m s)], steady static pressure and the lack of refined boundary mesh [33–35]. The inflow flow is the accepted difference between the normal and HF patients [4, 36–40], and there is no study to show that there are other factors to affect the PM2.5 movements and depositions in HF patients, so the assumption of the same upper airway structure is made. These assumptions have been accepted and widely used [2–10]. The specified velocity (or flow rate) reported in literature was constant, including 15, 30, and 60 l/min. In fact, in order to performance the breathing characteristics in patients with heart failure, the breathing velocity is individualized according to the change of the cardiovascular system function in patients with heart failure.

The rate of deposition and escaped is necessary to discuss the air flow state in the respiratory tract. For the patients with HF in this study however, the particle deposition rate is low, and the escape rate is high, so the particles have a much higher likelihood into the lungs. As we can see from Fig. 5, this is due to HF patients with inhalation of high value (949 > 920 ml), and the respiratory cycle is short (18 > 16 bpm), causing the high slope of respiratory rate, so the possibility of escape of the PM2.5 particles flow increases. In the present study, the inlet flow was the only variable to impact the movement of PM2.5, and several factors such as the structure of upper respiratory tract and particle types were ignored. There are no studies to show these differences, so it could be the further study.

Wall shear stress has been considered an important factor. Figures 6 and 7 show that the WSS was higher in the entrance and bronchus divides of patients with heart failure,and the wall pressure and WSS were obviously higher in HF than the normal control. So the upper respiratory system of HF could bear more pressure and damage to make further deterioration of the patients. Figure 8 shows that the gas flow velocity is very high (18 m/s) in the throat. The results were unreasonable, and this is associated with the narrow structure of the larynx caused by rigid boundary. So the study just provide some qualitative analysis and guidance. This also inferred that the respiratory and cardiovascular system have inseparable relationship. So the study can be considered as a bridge to research the cardiovascular diseases by the respiratory state. It also can explain the clinical phenomenon that with the increased concentration of PM2.5, the morbidity and mortality of HF patients would rise [7–9].

Limitations for the present study should be pointed out as well. Firstly, the inlet flow measurement were not standard, because the fact velocity curve wasn’t been recorded. Secondly, the CFD results are not validated by the experimental measurement. Thirdly, the refined boundary mesh was not be verified. Fourthly, the wall of upper respiratory tract was set to be absolute-adsorption, so the particles could stop if it contact with the wall. Fifthly, the particles’ types and quantity distribution was not statistical analyzed. Finally, the environment temperature and the upper respiratory tract mucous membrane are not considered. These factors will be the next research objects of the project in combination with clinical trials and cell experiment.

## Conclusions

In order to clarify the movement and deposition of PM2.5 particles and the hemodynamic difference between patients with heart failure and normal person, numerical studies were conducted. Results demonstrated that under the breathing pattern of patients with HF, the aerodynamic and particles movement states are distinguishing. The probability of PM2.5 entered into the lungs is higher, and the location of particle deposition was distributed mainly over the upper respiratory tract. The HF also has a higher WSS at the bifurcation of the bronchus and throat. These regions are prone to develop respiratory disease, further affect the cardiovascular system. These results are consistent with the physical experiment results of the upper respiratory tract adsorption to the particles.

## Abbreviations

HF: heart failure
CFD: computational fluid dynamics
ARLA: aerosol research laboratory of Alberta
WSS: wall shear stress
SD: standard deviation
LRN: low-Reynolds-number
NYHA III: New York heart association level III
